# Comparison of the treatment results of knee osteoarthritis using adipose tissue mesenchymal stromal cells derived through enzymatic digestion and mechanically fragmented adipose tissue

**DOI:** 10.1097/MD.0000000000024777

**Published:** 2021-03-05

**Authors:** Anna Maria Krześniak, Kuba Radzimowski, Artur Stolarczyk

**Affiliations:** Medical University of Warsaw, Warszawski Uniwersytet Medyczny, Warsaw, Mazovia, Poland.

**Keywords:** adipose tissue, cartilage, knee osteoarthritis, mesenchymal stromal cells

## Abstract

**Introduction:**

Knee osteoarthritis is a common condition that affects daily functioning and decreases the quality of life. There are many ways of treatment depending on the stage of the disease. Advanced cases are qualified for arthroplasty, which is an extensive and demanding surgical procedure. Less advanced stages are treated in various ways: from rehabilitation, through oral and intra-articular pharmacotherapy, to surgical treatment (arthroscopy, osteotomy). Because surgical treatment is risky, scientists focus on less invasive therapeutic methods. The most valuable management is based on regeneration. Mesenchymal stromal cells (MSC) derived from the adipose tissue have a great regenerative and anti-inflammatory potential, therefore an attempt is being made to take advantage of them in knee osteoarthritis treatment.

The study aims to compare the clinical effects of treatment of knee osteoarthritis using adipose tissue MSC obtained by an enzymatic method with the outcomes of the therapy with the mechanically fragmented adipose tissue.

**Methods:**

One hundred adults with primary knee osteoarthritis will undergo lipoaspiration under sterile conditions. The collected lipoaspirates will be further processed, depending on the randomly assigned group-enzymatically with the use of collagenase or mechanically using the Lipogems system. The preparations will be administered to the patients’ knee joints in the operating room under ultrasound control.

The results of treatment will be assessed using Knee Injury and Osteoarthritis Outcome Score, measuring the flexibility of the knee joint, evaluating joint gap in X-ray and the quality of cartilage in magnetic resonance T2-mapping during 1 year after treatment.

**Discussion/conclusion:**

Identification and functional analysis of the regenerative capacity of adipose-derived MSC depending on three variables (body weight, sex, and age) will help to develop a targeted therapy for different groups of patients and will determine the effectiveness of both methods of treatment. An attempt will be made to identify groups of patients with the greatest regenerative potential of the adipose tissue, and thus indicate those with the most probable improvement of the joint condition.

**Trial registration:**

This study protocol has been approved by the Ethics Committee of Medical University of Warsaw and registered on www.clinicaltrials.gov: NCT04675359 (06 Jan 2021)

## 1 Introduction

Knee osteoarthritis is one of the basic diseases in orthopedics. It impairs the function of the joints in about 10% of people over 55 years of age; in 25% the condition causes severe disability.^[1]^ The disease cannot be cured, but only slowed down. Joint pain and stiffness effectively hinder daily functioning. Cases of advanced osteoarthritis are qualified for arthroplasty, which is an effective but permanently mutilating procedure, and therefore it is considered a last resort. Less advanced stages of the disease are treated in various ways: from rehabilitation, through oral and intra-articular pharmacotherapy, to surgical treatment (arthroscopy, osteotomy). Because surgical treatment is burdensome and risky, it is worth focusing on less invasive therapeutic methods. The most valuable management is based on tissue regeneration. Following the trend in osteoarthritis research, regenerative medicine seems to be the treatment of the future.

The adipose tissue is considered the best source of mesenchymal stem cells (MSC) – there are many more MSC than in the bone marrow,^[2]^ they are easy to collect by lipoaspiration (colloquially liposuction). MSC are multipotent cells having mainly anti-inflammatory, immunomodulating, and trophic activity.^[3]^ The attempts are made to use the cells in various fields of medicine. Most of the research has been carried out in vitro and on animals. Scientists have been interested in the topic for over 20 years; in the last few years, the first preliminary reports on using human adipose-derived mesenchymal stromal cells (AD-MSC) appeared. For instance, the administration of the cells to the joints together with platelet-rich plasma is applied to reduce pain.^[4]^ A similar experiment was based on injecting AD-MSC of different densities to arthritic joints. The results of the self-assessment questionnaire revealed a reduction in pain and improvement of the joint function, as well as a decrease of the size of cartilage defects and increase the volume of cartilage in arthroscopic images, while the histological examination showed hyaline-like cartilage regeneration in patients who received the highest dose of cells.^[5]^ No side effects were reported. Another attempt to use these cells in humans applies to meniscal tears. A case of a healed meniscus tear in an adult was published with a significant decrease of pain after the AD-MSC injection in the knee joint.^[6]^ There were also satisfying results of the use of AD-MSC in the first carpometacarpal joint with osteoarthritis. Significant decrease of pain and improvement of hand function were achieved.^[7]^

Despite the growing interest in MSC, the knowledge about them still needs to be completed. More scientific studies, both laboratory and on humans, are necessary to explore the subject. Great potential of AD-MSC proven in vitro requires a clinical application. Cell therapies might be the answer to current problems in many diseases, including osteoarthritis, which leads to disability by a gradual deterioration in everyday functioning. Nowadays there are two major ways of processing adipose tissue available: enzymatic digestion to obtain stomal-vascular fraction (SVF) rich in MSC and mechanical fragmentation, rinsing and clarification of the tissue to create a product easy to inject, which is less purified but much easier to implement.

## 2 Materials and methods

### 2.1 Study design

The study will be carried out in the Orthopedics and Rehabilitation Clinic of Medical University of Warsaw. Based on the calculation of the sample size using power analysis of the test, investigators plan to include 100 adults with primary knee osteoarthritis in this study. The patients will undergo lipoaspiration under sterile conditions of the operating theatre, the collected lipoaspirates will be further processed depending on the randomly assigned group:

- enzymatically with the use of collagenase, or
- mechanically using the Lipogems system.

After collection, lipoaspirates will be immediately transferred to the laboratory of the Department of Regenerative Medicine with a tissue and cells bank in Warsaw, and the preparations will be administered to the patients’ knee joints in the operating room under ultrasound control.

### 2.2 Inclusion/exclusion criteria

Inclusion criteria:

- Adults with symptomatic gonarthrosis diagnosed based on the X-ray, MRI, and clinical examination, previously unsuccessfully treated (rehabilitation, NSAIDs, hyaluronic acid, and platelet-rich plasma).
- Primary knee osteoarthritis.
- Willingness and consent to participate in the study.
- Readiness to attend control visits.

Exclusion criteria:

- Failure to meet the inclusion criteria.
- Active chronic infection.
- Metal implant made of a ferromagnetic material that will exclude the patient from magnetic resonance imaging.
- Use of anticoagulants.
- General and intra-articular use of steroids during the last year.
- Pregnancy and breastfeeding.
- Any autoimmune and rheumatic disease, or conditions that influence the accuracy of test results.
- Taking drugs or cytostatic medications in the last 30 days.
- Mental disorders, alcohol, and drug addiction.

### 2.3 Interventions

The aim of the study is to compare the clinical effects of treatment of knee osteoarthritis using adipose tissue MSC obtained by an enzymatic method (collagenase digestion) with the outcomes of the therapy with the mechanically fragmented adipose tissue (using Lipogems system).

Lipogems system is defined as a minimal tissue manipulation system. It is a sterile single-use medical device intended for the closed-loop processing and transferring of autologous adipose tissue in a single surgical step.

In contrast, the administration of preparations produced using a collagenase digestion requires advanced actions in the laboratory of the tissue and cells bank, in which the specimens of SFV are prepared.

Both interventions demand adipose tissue collecting. Lipoaspiration will be performed using blind cannulas and vacuum syringes introduced through the abdominal wall with a 3-mm incision on the skin after previous injection to the adipose tissue with modified Klein's solution (500 ml of saline solution, 1 ml of adrenaline, and 40 ml of 2% lignocaine), which makes the collection easier. 10 ml of the collected adipose tissue will be used to examine the samples after previous gradient drainage from Klein's solution. Immediately after collection, the samples will be sent to the laboratory for analysis (cell count testing, culture, phenotype identification, and analysis by flow cytometry).

### 2.4 Outcome measurements

The patients will be monitored in the hospital outpatient clinic for 1 year after the procedure; the control visits will take place: after1, 3, and 6 months and after a year. A physical examination and the assessment using the Knee Injury and Osteoarthritis Outcome Score (KOOS) will be performed. Before the procedure and after 1 year, an X-ray and MRI with T-2 mapping will be carried out to evaluate the condition of the cartilage.

Primary outcome measures:

- Changes in patients reported outcome measures –KOOS

The KOOS is self-administered and assesses five outcomes: pain, symptoms, activities of daily living, sport and recreation function, and knee-related quality of life. The KOOS's five patient-relevant dimensions are scored separately: pain (nine items); symptoms (seven items); ADL function (17 items); sport and recreation function (five items); quality of life (four items). A Likert scale is used and all items have five possible answer options scored from 0 (no problems) to 4 (extreme problems) and each of the five scores is calculated as the sum of the items included.

Scores are transformed to a 0-100 scale, with zero representing extreme knee problems and 100 representing no knee problems as common in orthopaedic scales and generic measures. Scores between 0 and 100 represent the percentage of total possible score achieved.

[Time frame: 1, 3, 6, and 12 months after procedure]

- Changes in the flexibility of the knee joint

Range of motion assessment by goniometer measurements

[Time frame: 1, 3, 6, and 12 months after procedure]

Secondary outcome measures:

- Changes in X-ray imaging of the knee joint in a standing position (AP view)
  Articular cartilage volume evaluation by joint gap measurements
  [Time frame: 6 months and 1 year after procedure]
- Changes in MRI with T-2 mapping of the knee cartilage
  Articular cartilage quality evaluation in T-2 mapping
  [Time frame: 6 months and 1 year after procedure]

### 2.5 Statistical analysis

Results will be expressed as the standard deviations and means. Through utilizing the T-test or Mann-Whitney *U* test, according to normality distribution assessed by Shapiro-Wilk test, the analysis of continuous variables will be carried out. All the statistical analyses will be performed via the software of Statistica. *P*-value less than .05 indicates that there is statistical significance.

## 3 Discussion

Currently, the therapy of osteoarthritis is a serious problem because the initial stage of the disease is treated with oral medications, and the advanced stage is qualified for arthroplasty. Intermediate stages do not respond to standard methods of treatment, but they are also not eligible for surgery. Therefore, these patients are doomed to a worse quality of life due to pain and decreased activity. The therapy with mesenchymal stem cells derived from the adipose tissue can solve this problem. There have been already developed new ways to obtain the cells – enzymatic methods and mechanical processing. However, the characteristics of the patients who would benefit most from this therapy remain unknown. Apart from an improvement of the function of joints and a reduction of pain in patients with osteoarthritis, the authors will make an attempt to identify people whose adipose tissue has the greatest regeneration potential and can improve the condition of the joints. This management will facilitate making a decision to introduce this type of therapy in the future, having only basic information about patients; it will also help in choosing the method of treatment.

## Author contributions

**Conceptualization:** Anna Maria Krześniak.

**Data curation:** Anna Maria Krześniak.

**Methodology:** Anna Maria Krześniak, Kuba Radzimowski.

**Project administration:** Anna Maria Krześniak.

**Supervision:** Artur Stolarczyk.

**Writing – original draft:** Anna Maria Krześniak.
